# Quality of Life among Pediatric Neurocognitive, Speech, and Psychomotor Rehabilitation Professionals during the COVID-19 Pandemic: A Longitudinal Study on an Italian Sample

**DOI:** 10.3390/clinpract11040101

**Published:** 2021-11-15

**Authors:** Vincenza Cofini, Valeria Bianchini, Mario Muselli, Danila Budroni, Loreta Tobia, Giovanna Letizia Calò, Leila Fabiani, Stefano Necozione

**Affiliations:** 1 Department of Life, Health and Environmental Sciences, University of L’Aquila, Piazzale Salvatore Tommasi 1, 67100 Coppito, Italy; valeria.bianchini@univaq.it (V.B.); mario.muselli@graduate.univaq.it (M.M.); loreta.tobia@univaq.it (L.T.); giovannaletizia.calo@student.univaq.it (G.L.C.); leila.fabiani@univaq.it (L.F.); stefano.necozione@univaq.it (S.N.); 2 SanStefar Abruzzo, Rehabilitation Center, Via Basilicata 12, 64026 Roseto Degli Abruzzi, Italy; danila.budroni@gmail.com

**Keywords:** children rehabilitation, COVID-19 outbreak, health related quality of life, occupational stress

## Abstract

Objective: The aim was to estimate the perceived quality of life and its relationship with sociodemographic and professional factors, perception of susceptibility to COVID-19, and stress. Design: It was a longitudinal study. Subjects: Professionals, working in Italian centers for pediatric neurocognitive, speech, and psychomotor rehabilitation. Methods: Participants were interviewed online twice during the COVID-19 outbreak in Italy. The questionnaire included: (i) The measures of health-related quality of life to perform the Summary Index of Unhealthy Days, (ii) modified items from the “Standard questionnaire on risk perception of an infectious disease outbreak” and (iii) the items of the General Health Questionnaire. Results: One hundred and thirty professionals out of 130 participated in the first interview, while only 50 therapists took part in the second interview (dropout rate: 61%). The Summary Index of Unhealthy Days was 8 days at the first interview, and it decreased to 6 days at the second interview; however, the reduction was not significant (F = 3.22; *p* = 0.079). The multivariable analysis showed that the rehabilitation providers with moderate or severe stress level were more likely to have a negative perception of the quality of life (ORadj = 7.155; 95% CI: 2.8–18.2), and this result was confirmed at the second interview. Conclusions: Our results showed that in a severe public health emergency, the mental health and quality of life of rehabilitation professionals must be a topic of focus to enhance psychological resilience, to prevent burnout and to reduce rehabilitation errors.

## 1. Introduction

Due to the outbreak of SARS-CoV-2, the Italian Government imposed a national lock-down from March to May 2020, restricting population mobility and deferring health services delivery [1,2]. After the lockdown, to face the COVID-19 pandemic, the Italian healthcare system rapidly restructured its organization [3].

The whole health sector has been classified as at high risk of SARS-CoV-2 infection [4]. Rehabilitation professionals are also at high risk, in particular when they work with younger patients with disabilities. This category of patients, often affected by cognitive and behavioral disorders, may pose unique challenges for rehabilitation providers in ensuring social distancing and the use of personal protective equipment, with a possible greater risk of spreading the virus and transmission to healthcare workers. Thus, health services were redesigned, planned care was rescheduled, and the therapeutic set-up was modified to avoid all those rehabilitation modalities that involved direct contact between the patient and the professional [4]. In such situations, rehabilitation therapists, such as healthcare workers, may experience increased emotional and behavioral health concerns to a greater degree than the public [5]. Evidence from recent surveys indicates that since the outbreak of the COVID-19 pandemic, there has been a significant increase in mental health concerns such as depression, stress, anxiety, suicidality, sleep disorders, and substance use [6].

Interestingly, there are individual differences in susceptibility to mental health issues, including affective and physiological risk factors, as well as protective factors such as perceived social support, self-efficacy, and dispositional mindfulness [7].

Current literature reports that frontline healthcare workers have proven to be at increased risk of developing psychological symptoms and mental health disorders: increased workload, physical pressure, isolation and loss of social support, inadequate protective measures, professional viral transmission, and unprecedented ethical concerns on the rationing of care may have important consequences on personal physical and mental well-being of health professionals [8,9].

In countries across the world, the COVID-19 pandemic has led to heightened anxiety, depression, stress, and insomnia among healthcare practitioners. The current COVID-19 pandemic is characterized by some relevant features that increase the risk for PTSD among frontline healthcare workers due to the unprecedented numbers of critically ill patients, with an often-unpredictable course of the disease, high mortality rates and lack of effective treatment or of treatment guidelines, and the high risk of transmission [10].

With the increase in mental health symptoms and acute stress among healthcare workers, there has been a call to understand how this subgroup may be coping during the current global pandemic [11], including using behavioral and cognitive strategies [12]. Interestingly, healthcare workers have been found to enact lower levels of coping strategies compared to the public [13], despite other research findings suggesting that health professionals are interested in addressing their concerns about pandemic-related stress.

Several studies have been published to appeal to the mental burden of medical and nurse staff, to propose guidelines and to describe serious psychological disturbances, the most common of which were depression, anxiety, insomnia, fear, and burnout syndrome [14,15,16,17].

Few studies have described the impact of COVID pandemic on rehabilitation professionals.

To our knowledge, a previous study was carried out among Italian Rehabilitation professionals before the COVID-19 outbreak, and it showed burnout and work-related stress [18]. A recent study that included physicians, physical therapists, and nurses working in the Respiratory Intensive Care Unit, Neurology Unit, and Rehabilitation Unit showed a greater risk for depression, anxiety, and stress during the COVID-19 pandemic [19].

We focused our attention on rehabilitation professionals working in a pediatric setting for mental and cognitive intervention.

In Italy, during the lockdown, the pandemic forced changes in the organization of the health care services and assistance for children with disabilities, also considering the strong emotional stress and problematic behaviors put in place by children, caused by the rules for the prevention of contagion [20,21,22].

We hypothesized that quality of life perceived by rehabilitation professionals returning to the face-to-face care model after the lockdown, after adjusting for potential confounders including demographic factors, could be related to perceived stress and perception of susceptibility to the COVID-19.

This is the first study to evaluate health-related quality of life (HRQOL) measures COVID-19 during the COVID-19 outbreak in Italy.

Thus, we investigated the quality of life and its relationship with sociodemographic and professional factors, perception of susceptibility to COVID-19 and stress, on speech therapists, neuro- and psychomotor therapists and psychologists working in pediatric cognitive rehabilitation during the COVID-19 pandemic.

## 2. Materials and Methods

This was a longitudinal study conducted on a sample of rehabilitation professionals: speech therapists (SP), neuro- and psychomotor therapists (NPT), and psychologists (PT) working in health centers for child neurocognitive, speech and psychomotor rehabilitation.

The study was authorized by the Internal Review Board of the University of L’Aquila as part of the project “Knowledge, attitudes, perception of the risk of COVID-19 infection in university students and workers with different degrees of risk” (protocol code 79234/2020).

Two researchers, a psychologist and a psychiatrist, introduced the project to the target population in a web meeting and the questionnaire was promoted through mailing lists.

The participants were interviewed online after they gave their consent. No personal identifiers appeared in the database.

The inclusion criteria were:

(i). Working in public or accredited private rehabilitation centers for neuropsychiatric disorders of childhood;
(ii). Giving the informed consent.

The exclusion criteria were:

(i). No informed consent;
(ii). Not working in public or accredited private rehabilitation centers for neuropsychiatric disorders of childhood.

The interviews were planned after the Italian lockdown (6 months from the start of the COVID-19 outbreak) and approximately after 1 year from the outbreak, which corresponded to the second wave of COVID-19 pandemic in Italy.

### 2.1. Measures

Participants were interviewed online using a questionnaire composed of three modules based on three standard questionnaires:

(1) Self-perceived health and quality of life (HRQoL) questionnaire, developed by the Centers for Disease Control and Prevention and used in Italian Epidemiological Surveillance System in adult people (PASSI) [23,24,25]. The survey was based on the answers to four core questions:

I. How is your health in general? (Excellent, Very good, Good, Fair, or Poor).
II. Now, thinking about your physical health, which includes physical illness and injury, how many days during the past 30 days was your physical health not good?
III. Now, thinking about your mental health, which includes stress, depression, and problems with emotions, how many days during the past 30 days was your mental health not good?
IV. Now, thinking about your usual activities. During the past 30 days, approximately how many days did poor physical or mental health keep you from doing your usual activities, such as self-care, work, or recreation?

The first HRQOL measure on self-rated health status was dichotomized into fair or poor health and, good, very good, or excellent health. The two HRQOL questions on physical and mental health were combined to calculate the “Summary Index of Unhealthy Days”, with a maximum of 30 days. The Summary Index of Unhealthy Days is a validated index of self-reported mental and physical health that allows researchers to examine trends in health over time and identify groups of people that may need attention [26].

(2) Standard questionnaire on risk perception of an infectious disease outbreak adapted to COVID-19 [27]. For the present study we analyzed: the perception of susceptibility to the disease with the item “Do you think that you can contract COVID-19 in the coming week if you do not take any preventive measures?” rated on a 5-point Likert scale of agreement (from strongly disagree to strongly agree).

(3) General Health Questionnaire (GHQ-12) consisting of a 12-item scale used to assess perceived psychological distress: each item assessing the severity of a mental problem over the past few weeks using a 4-point scale (from 0 to 3). The GHQ-12 score ≤ of 15 was used to indicate an average stress level, a 15–20 score indicates a moderate level of stress, and a score ≥ of 20 indicates a more intense psychological distress [28].

The questionnaire also included items on socio-demographic information, training on preventive measures and implementation of the identified measures. Data were collected from June to July 2020 (first interview) and from November to December 2020 (second interview), during the second wave of the COVID-19 pandemic in Italy.

### 2.2. Outcomes

The first outcome was estimating the perceived quality of life and its relationship with the demographic factors, rehabilitation categories, training, perceived stress, and perception of susceptibility to the COVID-19. We also investigated the factors associated to withdrawal from the study.

### 2.3. Statistical Analysis

This was not a representative sample of the population of SP, NPT, and PT working in child speech therapy, neurocognitive, and psychomotor rehabilitation centers, but it was a convenience sample, and a formal sample size calculation was not performed. One hundred and fifty rehabilitation providers participating in three web seminars on the topic “COVID and rehabilitation” were invited to the study.

All variables were analyzed and reported as frequencies or mean and standard deviation (SD) or median and IQR, if they were qualitative or quantitative variables, respectively. A repeated-measures Anova model was used to compare mean values. All analyses were carried out with the STATA 14 software, setting alpha to 0.05. To perform univariate and multivariable logistic analysis, variables such as the Summary Index of Unhealthy Days, age, perceived stress, and perception of susceptibility to the SARS-CoV-2 were dichotomized on the basis of median value. The models were run using the Summary Index of Unhealthy Days (≤4 vs. >4), as dependent variable; gender (males vs. females), age, civil status (single or divorced vs. married or partner), rehabilitation category (SP/SNP/PT), perceived stress (≤18.5 vs. >18.5), perception of susceptibility to the COVID-19 (≤4 vs. >4), training (yes/no) as independent variables. Adjusted odds ratios (ORadj) with 95% confidential intervals (CI) were reported for all the investigated variables.

## 3. Results

### 3.1. Inclusion/Exclusion Results

**Figure 1:**
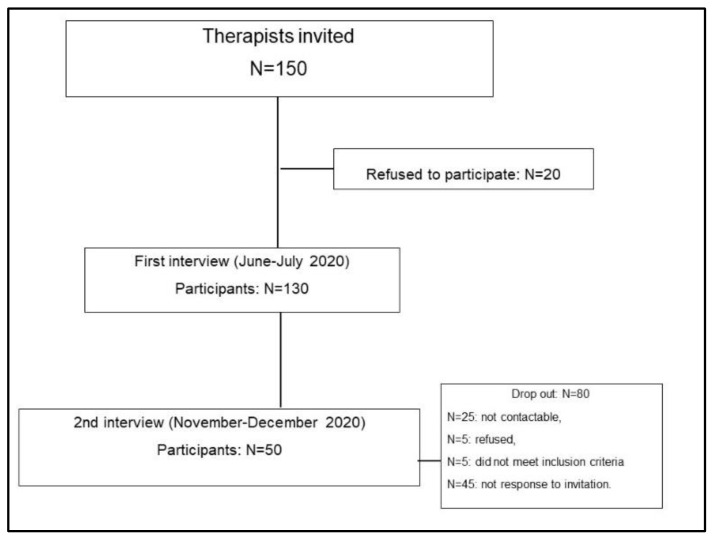
The study flow chart.

One hundred and fifty rehabilitation providers participating in three web seminars on the topic “COVID and rehabilitation” were invited to the study. One-hundred and thirty subjects (87%) were asked to fill in the anonymous questionnaire at the first interview (after the lock-down). Only 50 subjects participated in the second interview, (during the second Italian wave of COVID-19 pandemic). Eighteen rehabilitation providers dropped out of the study, and the dropout rate was about 61% (Figure 1).

### 3.2. Characteristics of Participants

All the participants were informed about the preventive measures to contain the spread of the virus and to protect themselves and patients during rehabilitation care. The preventive measures of COVID-19 were correctly carried out “always” by 91% of participants.

Table 1 reports the participants’ background parameters at the first and second interviews: The participants were prevalently females: 116/130 (89%) at the first interview and 37/50 (74%) at the second interview, with a mean age of 36 years (10.5) and 41 years (10.8), respectively. Seventy-one percent (93/130) reported receiving some specific training on COVID-19 preventive measures at the first interview, and 84% at the second interview.

### 3.3. Survey Results

As reported in Table 2, at first interview, fifty-three out of 130 participants reported an excellent health status (41%; 95% CI: 33–50%), and 58% had a good or very good health status (95% CI: 49–67%) with only one participant reporting a fair health status (1%). Overall, the rehabilitation providers perceived 2.4 physically unhealthy days in the past 30 days, while they reported 6 mentally unhealthy days for 2 days per month, their activities were limited due to poor self-perceived physical and mental health status. The Summary Index of Unhealthy Days for all participants was 8 days (SD = 9) (95% CI: 7–10), and the median value was 4 (IQR: 11–9).

At the second interview, thirty-three out of 50 participants reported a good or very good health status (95% CI: 49–67%) and 16 had an excellent health status (95% CI: 6–18%), and only one rehabilitation provider perceived a fair health status (2%). The rehabilitation professionals perceived on average 1.6 physically unhealthy days in the past 30 days, while they reported 4.3 mentally unhealthy days. The Summary Index of Unhealthy Days was 6 days (SD = 6) (95% CI: 4–8).

**Figure 2:**
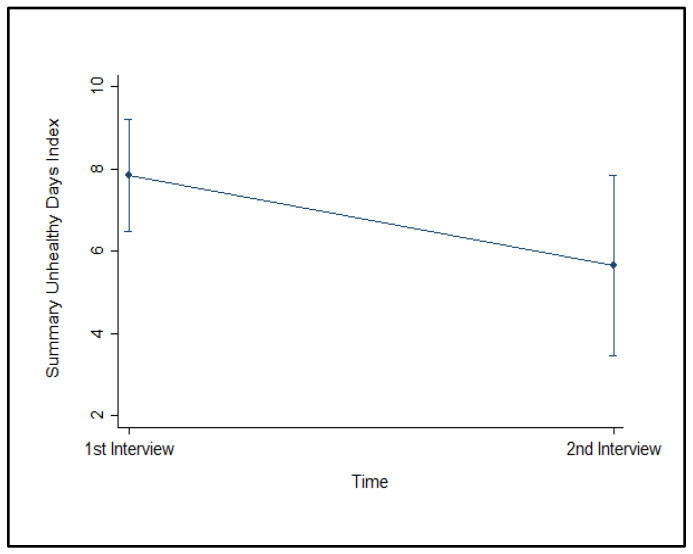
Summary Index of Unhealthy Days, (mean) over time.

The Summary Index of Unhealthy Days was 6 days (SD = 6) (95% CI: 4–8), and the reduction from the first evaluation was not significant (F = 3.22; *p* = 0.079), as reported in Figure 2.

**Figure 3:**
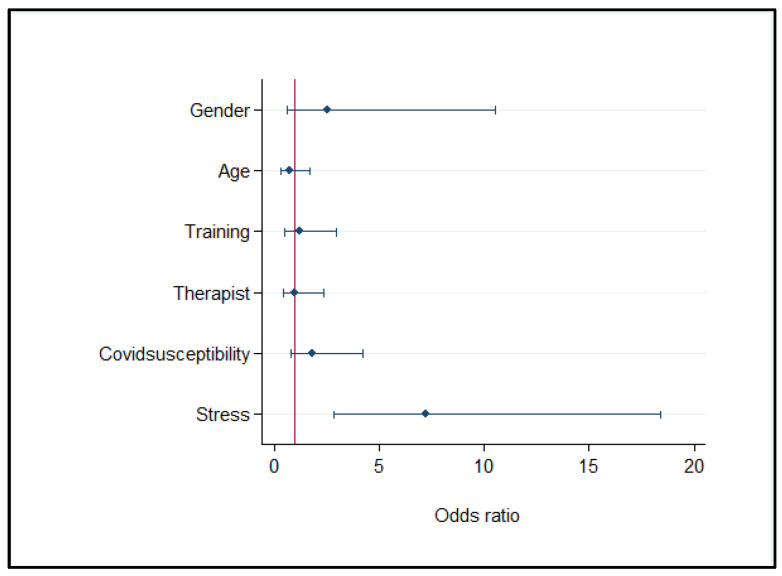
Factors associated to the Summary Index of Unhealthy Days (≤4 vs. >4 days), Multivariable logistic regression model (first interview: *n* = 130).

The multivariable analysis on the first interview responses showed that the perceived stress was the factor associated to the Summary Index of Unhealthy Days. As reported in Figure 3, the rehabilitation providers with moderate or severe stress level were more likely to perceive their quality of life negatively, that is seven times higher than rehabilitation providers without stress (ORadj = 7.155; 95% CI: 2.8–18.2).

The multivariable logistic regression on data of the second interview, showed that the only factor statistically related to the unhealthy days was the perceived stress (ORadj = 45.731; 95% CI: 4.55–459.83).

## 4. Discussion

This was a longitudinal study conducted on a sample of Italian speech therapists (22%), neuro- and psychomotor therapists (42%) and psychologists (35%) working in pediatric rehabilitation during the COVID-19 pandemic.

One hundred and thirty participants were interviewed after the necessary reorganization of health services to protect the health and safety of patients and workers. The aim of the study was to investigate the perceived quality of life and its relationships.

It is known that among health workers the quality of life could be related to their work and therefore to the patients’ response [29].

At the first interview, 58% of the participants reported a good or very good health status and the proportion increased to 66% at the second interview. These results appeared to be lower than the 2016–2019 data from Italian Surveillance System PASSI that used the same methodology to assess the perceived health status and quality of life [30]. The Passi System, in fact, showed that 89.7% (95% CI: 89.3–90.1%) of people aged 18–34 and 76.2% (95% CI: 75.7–76.7%) of people aged 35–49 perceived their health status as very good or excellent.

With respect to the Summary Index of Unhealthy Days (physically and mentally unhealthy days), the study reported that the rehabilitation providers perceived, on average, 8 unhealthy days at first interview (95% CI: 7–10), and 6 months later they reported on average 6 unhealthy days (95% CI: 4–8), which was higher than the 4.4 days reported by the Passi System (95% CI: 4.3–4.4) [30].

Our findings might be in line with the fact that working in very specific services and in situations of extreme vulnerability affects professionals’ mental health [31]. Rehabilitation professionals were on the frontline as being in physical and psychological contact with the patient, especially with the younger patients with disabilities and unable to maintain adequate physical distancing and COVID-19 mitigation measures. Children with neurodevelopmental disorders, in fact, may encounter difficulties in adapting to abrupt changes. This is mostly found in diseases such as attention deficit and hyperactivity disorder and autism spectrum disorder in which children show important limits in emotional and cognitive self-regulation, manifesting difficulties in respecting social rules, in adapting to emotionally difficult situations and inhibiting inappropriate behavior. This can often lead to irritability in patients, provocative or aggressive behaviors towards the therapist [32].

The multivariable analysis revealed that the Summary Index of Unhealthy Days was significantly related to perceived stress over time. The difficulty of the face-to-face rehabilitation for the disabled patients could have been an important stressor in the complex health environment during the pandemic. Our study is consistent with the results of other studies that reported how work-related stress results in greater perceptions of poor physical and mental health [33,34].

Previous research showed a high level of stress and psychological distress among healthcare workers in the first month after the pandemic outbreak, without differences between persons that worked in COVID-19 wards versus those working in non-COVID-19 wards [35]. Over time, several studies evidenced a higher psychological impact among frontline healthcare providers compared to non-frontline medical workers [36].

Our study has some limitations. The sample was unbalanced by job category and by gender, with a higher presence of women. Although the sample was representative of the gender distribution of Italian healthcare workers, it could not be considered representative of the different pediatric rehabilitation specialties [37,38]. Certainly, the study presents some other limitations, mainly related to the online convenience sample, to the lack of a priori sample size estimation, to the drop-out rate, to the lack of information about the engaged time or time periods for the patients during the COVID pandemic, and to the lack of psychological evaluation of the participants and their coping strategies.

As reported in literature, during the COVID-19 pandemic, much research has been done through convenience sampling using online questionnaire administration [8].

As known, the problem of the dropouts may be a significant source of bias. In general, in longitudinal surveys it may be related to three separate sources, that is failure to locate research participants, failure to contact participants, and failure to achieve cooperation [39].

We would like to emphasize that when the research was initially conducted the participants were exiting lockdown and they were returning to work, with the implementation of epidemiological measures to prevent COVID-19. At the time of the second interview, the participants were experiencing the second wave of the COVID-19 pandemic and we do not know if among the reasons for dropout, could be their possible coronavirus infection.

Burnout was a major issue faced by health care professionals in the pandemic [40]. Our survey may have overestimated the quality of life because a high percentage of individuals who are either burned-out or on the road to burnout, usually have a lower level of engagement and response to the surveys.

The study was based on a small convenience sample of professionals, therefore the lack of sample size estimation, as well as the possible selection bias related to recruitment, limited the generalizability of the results since we cannot be certain that the respondents were not systematically different from those non-responding. The effects of the high drop-out rate may also impact the generalizability and the statistical power. Disregarding the possible biases related to the high drop-out rate and the other limitations of the study, we may draw some important conclusions from our data, considering that the study was conducted during the Italian COVID-19 pandemic.

Moreover, the measures were repeated over time during the COVID-19 pandemic, and no previous research has examined the HRQOL measures among rehabilitation professionals. Thus, further research is needed to explore and understand how to assist rehabilitation practitioners in better managing their work-related stress during a major public health crisis and its impact on their life. Healthcare workers have been recognized as the occupational category most involved in risk infection and their dual role as victims and vectors must be highlighted [41]. The research shows that during the COVID-19 pandemic, rehabilitation professionals have been exposed to high levels of emotional distress, of risk perception and of fear for their own health and for the patients. It would be desirable to encourage communication within the team, to monitor the state of well-being and job satisfaction among healthcare workers to avoid the risk of burnout and to improve the quality of work for both the professional and the patient.

## Figures and Tables

**Table 1:** Participants’ background parameters at the 1st and 2nd interviews.

Characteristics	1st Interview (June–July 2020) *n* = 130	2nd Interview (November to December 2020) *n* = 50
	Mean (SD) or *n* (%)	Mean (SD) or *n* (%)
Age (years)	36 (10.5)	41 (10.8)
Gender	-	-
Female	116 (89%)	37 (74%)
Male	14 (11%)	13 (26%)
Residence	-	-
Northern Italy	35 (27%)	4 (8%)
Central Italy	59 (45%)	24 (48%)
Southern Italy	36 (28%)	22 (44%)
Civil status	-	-
Married	50 (38%)	23 (46%)
Civil partner	17 (13%)	9 (18%)
Divorced	6 (5%)	2 (4%)
Single	57 (44%)	16 (32%)
Health rehabilitation category	-	-
Speech therapy	31 (22%)	7 (14%)
Psychology	47 (36%)	33 (66%)
Neuro-psychomotricity	82 (42%)	10 (20%)
Training on COVID-19	-	-
Yes	93 (71%)	42 (84%)
No	37 (29%)	8 (16%)

**Table 2:** Quality of life, perception of susceptibility to COVID-19, perceived stress by time.

Variables	1st Interview *n* = 130	2nd Interview *n* = 50
	Mean (SD) or *n* (%) (95% CI)	Mean (SD) or *n* (%) (95% CI)
How is your health in general?		
Excellent	53 (41) (33–50)	16 (32) (20–46)
Good or very good	76 (58) (49–67)	33 (66) (51–78)
Fair or Poor	1 (1) (0.1–5)	1 (2) (0.2–13.7)
Physical unhealthy days	2.4 (5) (1.7–3.1)	1.5 (2) (0.4–2.7)
Mental unhealthy days	6 (5) (4.7–7.3)	4.3 (1) (2.2–6.3)
Unhealthy days with activity limitation	2 (3) (1.7–2.8)	1 (2) (1.0–2.4)
Summary Unhealthy Days Index	8 (9) (7–10)	6 (6) (4–8)
Perception of susceptibility to COVID-19	4 (0.9) (3.9–4.2)	4.3 (0.7) (4–4.5)
Perceived stress (GH-12 score)	18.7 (3.6) (18.1–19.3)	19.8 (3.2) (18.1–20.0)
